# Immunological and nutritional factors in elderly people in low-level care and their association with mortality

**DOI:** 10.1186/1742-4933-10-32

**Published:** 2013-08-05

**Authors:** Julie L Woods, Sandra Iuliano-Burns, Karen Z Walker

**Affiliations:** 1 Nutrition and Dietetics Department, Monash University, Melbourne, Australia; 2 Endocrine Centre of Excellence, Department of Medicine, Austin Health, University of Melbourne, Melbourne, Australia; 3 Department of Nutrition and Dietetics, Monash University, Level 1, 264 Ferntree Gully Road, Notting Hill, VIC 3168, Australia

**Keywords:** Aged care, Dietary intake, Lymphocyte subsets, Mortality

## Abstract

**Background:**

This study examines associations between markers of nutritional status and lymphocyte subsets and seeks to determine if lymphocyte profile is predictive of survival in elderly Australians residing in aged care facilities. Aged yet still ambulatory subjects (*n* = 88, 73% female) living in low-level care and requiring minimal assistance were studied for 143 weeks. At baseline when participants were aged (mean ± SD) 86.0 ± 5.9 years, dietary intake was determined by 3-day weighed food record, body composition was assessed by dual energy X-ray absorptiometry (DXA) and a venous blood sample was taken.

**Results:**

At baseline assessment, study participants were consuming nutrient-poor diets and most had symptoms of chronic disease. Although overweight, 40% exhibited sarcopenia. Markers of nutritional status did not relate closely to immune cell numbers (absolute or relative), which on average were within the normal range. Men had lower numbers of CD3^+^CD4^+^ cells (CD4^+^ T cells), a higher proportion of CD3^−^ CD16^±^ CD56^±^ (natural killer (NK) cells) and a higher ratio of NK: CD4^+^ T cells than women (all *P* < 0.05). The main age-related changes evident were decreased T cells, particularly low CD4^+^ T cell counts, and increased numbers of CD19^+^ (B-cell) and NK cells. During the 143 week duration of follow-up, about one quarter of the study participants died, with death more likely in men than women (*P* < 0.01). Poor survival was predicted by the presence of decreased numbers of CD4^+^ T cells (hazard ratio (HR) 0.919, *P* < 0.01) and expanded numbers of NK cells (HR 1.085, *P* < 0.05) in the blood, and therefore the presence of a high NK: CD4^+^ T cell ratio (HR 30.521, *P* < 0.01).

**Conclusions:**

The NK: CD4^+^ T cell ratio may potentially have clinical utility for predicting longevity in elderly populations. Further studies are needed in other elderly populations to confirm this finding.

## Introduction

Natural ageing is associated with a progressive deterioration of immune system function, described as i*mmunosenescence*[1] or *senescent immune remodelling*[2]. With aging, the innate system barriers become less able to restrict entry of pathogens [2], while changes in the numbers and/or function of innate and adaptive immune cell populations contribute to an increased risk of morbidity [3], poor responses to vaccination [4], and increased mortality [5], [6].

Natural aging causes many changes in the adaptive immune system. Involution of the thymus and deterioration in its internal architecture is accompanied by accumulation of adipocytes, defects in thymic epithelial cells and an inability to export many naïve T cells to the periphery [7]. Similar changes within bone marrow contribute to decreased lymphopoesis, so that cell populations skew towards myelopoesis [7], [8]. As with T cells, fewer naïve B cells are exported [2]. Clinically, the defects in immunity that appear with aging, lead to increased infection, increased risk of malignancies and higher rates of autoimmune disease [6].

The neutrophils, macrophages and natural killer (NK) cells of the innate immune system are also affected by aging. Activation and signalling by macrophages and neutrophils may become defective, and these cells can then exhibit decreased chemotaxis and phagocytosis and become less able to destroy internalised infectious organisms [9]. A redistribution of NK cell populations may also occur as numbers of immature NK cells decrease and more highly differentiated NK cells accumulate [10].

Good nutrition has a strong influence on immunity and is of particular importance in healthy aging [11]. Yet malnutrition is highly prevalent in institutionalised older people [12]– [15] as a result of poor appetite, reduced food intake, effects of medical conditions and medications, and even provision of nutritionally inadequate diets [14]. Although improvements in immune function with nutrient supplementation have been reported [16], [17], few studies have investigated the relationship between poor immune function and nutritional intake, serum nutrient status, body composition and/or other markers of poor nutrition such as serum albumin levels [18], [19].

This study describes the lymphocyte subset profile of elderly people in low-level aged care and investigates associations between markers of nutritional status and lymphocyte subsets. In addition, we examined the important question of whether lymphocyte profile is predictive of survival.

## Results

At baseline, participants were aged 86.0 ± 5.9 years (Table 1). There were no differences between men and women in relation to age or number of medical conditions and medications. Many participants suffered from chronic diseases, particularly cardiovascular disease (55%), lung disease (25%), renal disease (9%), diabetes (10%) and/or Parkinson’s disease (7%). One quarter had suffered a stroke. None had active cancer diagnosed by a general practitioner at the time of assessment although 15% had experienced cancer previously. There was no association between the presence of a chronic medical condition at baseline and participant age, or with any measures of body composition, the presence of sarcopenia, haematological parameters or dietary intake.

**Table 1:** Characteristics of the elderly study population (mean ± SD)

	**Women (*n*** **= 64)**			**Men (*n*** **= 24)**		
		**% below**	**% above**		**% below**	**% above**
		**Reference**^**d**^			**Reference**^**d**^	
Age (years)	85.9 ± 5.3			86.3 ± 7.5		
Medical conditions^a^	5.0 ± 2.2			5.4 ± 2.3		
Medications^b^	9.2 ± 4.4			9.5 ± 4.8		
Died (%)	16			42**		
Survival (weeks) ^c^	132.6 ± 3.3			109.0 ± 9.1 **		
***Selected dietary intake parameters per day***						
Energy (kJ)	6187 ± 1158	70		7887 ± 1573***	50	
Protein (g)	53.1 ± 10.9	21		69.7 ± 17.1***	41	
(% E)	14.7 ± 2.1			15.1 ± 2.6		
Fat (g)	58.4 ± 14.1			72.9 ± 15.5***		
(% E)	34.7 ± 3.5			34.3 ± 3.1		
Monounsaturated fat (g)	19.5 ± 4.7			24.3 ± 5.4***		
Polyunsaturated fat (g)	9.2 ± 2.8			10.2 ± 3.1		
Saturated fat (g)	25.0 ± 7.2			32.3 ± 8.9***		
Total retinol (μg)	1094 ± 320	1.8	59	1283 ± 335*	4.5	46
Folate (ug)	197 ± 60	96		258 ± 58***	86	
Vitamin C (mg)	122 ± 56		32	124 ± 42		41
Iron (mg)	8.1 ± 1.8	9		10.2 ± 2.7**		
Zinc (mg)	6.5 ± 1.3	52		8.6 ± 2.2***	91^**^	
Magnesium (mg)	201 ± 41	96		260 ± 75***	86	
***Selected body composition parameters***						
BMI (kg/m^2^)	26.6 ± 4.4	3.3	20	25.9 ± 3.4	4.3	13
Body fat (%)	37.8 ± 7.9			26.1 ± 5.8***		
SMI%	25.2 ± 3.7	41		32.0 ± 2.6***	45	
% with weight loss	59.6			56.3		
***Selected biochemical parameters***						
Albumin (g/L)	39.2 ± 3.4	8		38.1 ± 3.8	22	
Haemoglobin (g/L)	126 ± 12	8		131 ± 18	38^**^	
IL-6 (pg/mL)	4.15 ± 2.4		1.8	6.17 ± 33.6**		9.5
CRP (mg/L)	3.0 ± 2.5		40	8.7 ± 10.0*		67*

*P < 0.05, ** *P* ≤ 0.01, *** *P* < 0.001.

^a^Number of medical conditions present from those listed under Methods.

^b^Number of medications taken over the longer term. Medications for a temporary acute condition are not included.

^c^Until death. Data presented as mean ± standard error for this variable only.

^d^For dietary intake this is the % with intake below the estimated average requirement (EAR) for Australians or % above the recommended dietary intake (RDI) for Australians [45]; for BMI this is below 20 (kg/m^2^) and above 30 (kg/m^2^); for SMI% this is below 23.8% for women [44] and below 31.5% for men (unpublished but based on young male dataset as per [44]); for albumin this is the reference range provided by Network Pathology; for haemoglobin, CRP and IL-6 this is the reference range provided by Southern Cross Pathology.

Abbreviations: *BMI* body mass index, *CRP* C-reactive protein, *IL-6* Interleukin 6, *SMI (%)* Percentage skeletal muscle, *% E* percentage of total energy.

Elderly men exhibited a significantly higher dietary intake of most nutrients (*P* < 0.05) than elderly women (Table 1) but this difference was lost after correcting for differences in energy intake. Fifty percent of men and 70% of women had energy intakes below recommended levels. Many residents also had nutrient intakes below requirements, including (% men, % women): protein (41%, 21%), folate (86%, 96%), magnesium (86%, 96%), and zinc (91%, 52%).

On average, the elderly participants in this study were overweight as indicated by mean body mass index (BMI) (60% of women and 48% of men had a BMI > 25 kg/m^2^). Men retained more skeletal muscle as evidenced by a higher percent skeletal muscle (SMI%) (*P* < 0.001) and had proportionately less body fat (*P* < 0.001) than women. Despite the prevalence of adiposity, approximately 40% of men and women were classified as sarcopenic (by SMI%) and around 60% lost weight over the 143 week duration of the study. Mean haemoglobin and albumin levels were similar in both sexes, although a significantly higher proportion of men had low haemoglobin levels (*P* < 0.01). Men had higher mean levels of C-reactive protein (CRP) than women (*P* < 0.05) and more men had CRP elevated above the reference range (*P* < 0.05). Additionally, men had higher mean levels of interleukin-6 (IL-6) (*P* < 0.01).

Table 2 shows mean absolute and relative lymphocyte subset counts in the study population. Although average values for both men and women were within the normal range, over one third of women and nearly half of men had low CD19 (B-cell) numbers. Additionally, 50% of women and 65% of men exhibited low reactive oxygen species (ROS) production by neutrophils (data not shown). Men had a significantly lower CD4^+^ T cell count, a higher proportion of natural killer (NK) cells and a higher ratio of NK:CD4^+^ T cells than women (all *P* < 0.05). Additionally, more men than women displayed raised neutrophil counts. The mean CD4^+^:CD8^+^ ratio was less than 1.0 for only four individuals. No differences were evident in age, medical conditions, body composition, CD4^+^ T cells or survival rate between these individuals and those with a CD4^+^:CD8^+^ ratio > 1.0.

**Table 2:** Immune indices and lymphocyte subsets of the study population

	**Women (*n*** **= 64)**			**Men (*n*** **= 24)**			
	**Mean**	**% below**	**% above**	**Mean**	**% below**	**% above**	**Reference**
		**Reference**			**Reference**		**Range**
White blood cell count (×10^9^/L)	7.0 ± 1.6	1.6		7.7 ± 2.3		8.3	4–11
Total lymphocytes (×10^9^/L)	1.6 ± 0.4	4.7		1.5 ± 0.5	12.5		1.0–4.0
Neutrophils (×10^9^/L)	4.5 ± 1.3			5.1 ± 1.9		8.3^*^	2.0–8.0
Monocytes (×10^9^/L)	0.6 ± 0.2		6.2	0.7 ± 0.2		12.5	0.0–1.0
**CD3**^**+ **^**(total T cells)**							
Count (×10^6^/L)	1174 ± 338	6.2		1021 ± 355	16.7		688–2445
Percentage	73.5 ± 8.5	4.7	9.4	70.8 ± 7.8	8.3	8.3	59–84
**CD3**^**+**^**CD4**^**+ **^**(CD4**^**+ **^**T cells)**							
Count (×10^6^/L)	824 ± 279			697 ± 239*	8.3^*^		389–1569
Percentage	51.5 ± 10.5		18.8	47.1 ± 7.3		4.2	31–59
**CD3**^**+**^**CD8**^**+ **^**(CD8**^**+ **^**T cells)**							
Count (×10^6^/L)	329 ± 160	8		292 ± 151	21		168–894
Percentage	20.2 ± 7.9	12.5		20.1 ± 8.1	12.5		12–42
**CD4**^**+**^**:CD8**^**+ **^**ratio**	2.8 ± 1.4			2.8 ± 1.4			
**CD19 (B-cells)**							
Count (×10^6^/L)	145 ± 85	35.9		109 ± 73	45.8		98–597
Percentage	8.9 ± 4.6	29.7		7.3 ± 3.9	41.7		6–26
**CD3**^**−**^**CD16**^**+**^**CD56**^**+ **^**(NK cells)**							
Count (×10^6^/L)	266 ± 122	1.6		291 ± 129			61–776
Percentage	16.7 ± 6.5	4.7	6.3	20.2 ± 7.9*	4.2	12.5	7–28
**NK: CD4**^**+ **^**T ratio**	0.35 ± 0.20			0.45 ± 0.22 *			

*n* = 88 (mean ± SD).

*P < 0.0.

Age was negatively associated with absolute numbers of CD3^+^ cells (*r* = −0.209, *P* < 0.05) and both absolute and relative numbers of CD4^+^ T cells (*r* = −0.266, *r* = −0.218, respectively, both *P* < 0.05). Age was positively associated with the relative proportion of NK cells (*r* = 0.248, *P* < 0.05) as well as with the ratio of NK:CD4^+^ T cells (*r* = 0.245, *P* < 0.05). The number of medical conditions experienced by participants at baseline was negatively related to the proportion of CD4^+^ T cells (*r* = −0.215, *P* < 0.05) and positively related to the proportion of CD8^+^ T cells (*r* = 0.221, *P* < 0.05). In addition the number of long-term medications taken by participants was negatively related to the proportion of B cells (*r* = −0.230, *P* < 0.05) in blood but not to proportions of CD4^+^ or CD8^+^ T cell cells. Levels of CRP were negatively related to the proportion of CD8^+^ T cells (*r* = −0.235, *P* < 0.05).

By multivariate analysis the relative numbers of lymphocyte subsets were not explained by dietary intake (by any nutrient either in terms of total intake or as nutrient density), weight loss, BMI, SMI% or haemoglobin concentrations. However, after adjustment for age, gender and medical conditions (Table 3), relationships were evident between serum albumin and the relative numbers of both CD4^+^ T cells and B-cells (*P* < 0.05, *P* = 0.001 respectively) and between percentage body fat and proportion of B-cells (*P* < 0.05).

**Table 3:** Relationship between lymphocyte subset percentages and selected markers of nutritional status

**Measure**			**CD4**^**+ **^**T %**				**CD19%**	
**Total model R**^**2**^			0.200				0.232	
***P***			0.007				0.002	
**Independent variable**	**B**	***P***	**Variation explained**^**a**^	**CI**	**B**	***P***	**Variation explained**^**a**^	**CI**
Age (yrs)	−0.21	0.06	4.2	−0.72, 0.02	−0.05	0.64	0.2	−0.20, 0.13
Sex	−0.18	0.18	2.1	−9.75, 1.90	0.07	0.62	0.3	−1.94, 3.25
Medical conditions^b^	−0.22	0.43	4.9	−1.95, −0.03	0.01	0.89	0.0	−0.40, 0.46
Body fat (%)	0.06	0.66	0.2	−0.22, 0.36	0.30	0.02	6.1	0.02, 0.28
Albumin (g/L)	−0.27	0.02	7.0	−1.35, −0.14	0.37	0.001	13.7	0.21, 0.75

^a^Percentage of variation explained uniquely by the independent variable after adjustment.

^b^Medical conditions confined to those listed in Methods.

Total mortality (10 men, 10 women) was determined 143 weeks after study commencement. At this time 42% of the men had died but only 16% of women (P < 0.01). Men experienced death earlier than women (109.0 ± 9.1 weeks vs 132.6 ± 3.3, *P* < 0.01) (Table 1). Relative and absolute numbers of CD3^+^ cells, CD4^+^ T cells and B-cells were all unrelated to survival (Table 4). The presence of more NK cells (hazard ratio (HR) 1.085, *P* < 0.05) and fewer CD4^+^ T cells (HR 0.919, *P* < 0.01) in the blood was associated with a decreased chance of survival as was a higher ratio of NK: CD4^+^ T cells (HR 30.521, *P* < 0.01) (Table 4). All these relationships remained after adjusting for confounding variables.

**Table 4:** Relationship between risk of death and lymphocyte subset percentages (hazard ratios and 95% confidence intervals)

**Model**	**CD4**^**+ **^**T %**	**CD8**^**+ **^**T %**	**CD4**^**+**^**:CD8**^**+ **^**ratio**	**B cell %**	**NK cell %**	**NK:CD4**^**+ **^**T ratio**
No adjustment	0.939 (0.896, 0.985)**	1.004 (0.947, 1.065)	0.774 (0.545, 1.098)	0.938 (0.839, 1.049)	1.094 (1.026, 1.167)**	22.676 (3.492, 147.238)***
Age and sex	0.930 (0.881, 0.982)**	1.014 (0.953, 1.080)	0.726 (0.497, 1.061)	0.954 (0.852, 1.069)	1.084 (1.012, 1.161)*	23.568 (3.112, 178.459)**
Age, sex, medical conditions^a^	0.922 (0.869, 0.977)**	1.019 (0.954, 1.087)	0.712 (0.481, 1.054)	0.948 (0.844, 1.066)	1.085 (1.012, 1.163)*	27.132 (3.377, 217.978)**
Fully adjusted^b^	0.919 (0.863, 0.978)**	1.021 (0.955,1.091)	0.697 (0.452, 1.075)	0.982 (0.851, 1.133)	1.085 (1.008, 1.168)*	30.521 (3.151, 295.627)**

^a^Medical conditions confined to those listed in Methods.

^b^Adjusted for age, sex, medical conditions, body fat percentage and serum albumin level.

*P < 0.05, ** *P* ≤ 0.01, *** *P* ≤ 0.001.

## Discussion

This study describes an elderly, mildly overweight Australian population living in low-level aged care. Many residents were experiencing chronic diseases and despite the prevalence of overweight, many had nutrient-poor diets, and exhibited sarcopenia. Markers of nutritional status did not however relate closely to absolute or relative numbers of immune cells, which at study baseline, were found to be within the normal range. The main age-related changes evident were decreased T cells, particularly CD4^+^ T cells; increased B cell and NK cell numbers and the low production of ROS by neutrophils. These changes were seen in some but not all individuals. During the 143 week duration of the study, more than half these elderly people lost weight and around one quarter of them died. Men were more likely to die than women. Poor survival over this period was predicted by the presence of decreased numbers of CD4^+^ T cells and expanded numbers of NK cells in the blood, and therefore the presence of a high NK:CD4^+^ T cell ratio.

Malnourished elderly are known to have reduced immunity [20], [21]. Many elderly in our study had high dietary total retinol intake, a vitamin that may be important in protecting against Alzheimer’s disease [22]. A high dietary intake of vitamin A has previously shown to be associated with reduced T cell responses to the mitogen phytohaemagglutinin in community dwelling elderly [23]. Many elderly in our study also exhibited low intakes of dietary zinc. The importance of zinc in immune function is well established particularly for its role in maintenance of thymic function and in the production and export of T cells to the periphery [24]. Adequate levels of zinc may also be important in maintaining NK function [25]. The majority of elderly in our study also had low dietary magnesium. While less studied than zinc, this nutrient is also known to have a role in immunity, especially in the elderly where magnesium deficiency may accelerate thymic involution and increase inflammation [26].

Despite evidence for low dietary intakes of zinc and magnesium, clear associations between intakes of these nutrients and any specific immune deficit were not demonstrated in the present study. Nevertheless, previous studies have found that protein, iron and zinc levels in serum are predictors of immune function in elderly women [19]. One explanation for the discordance between our results in relation to dietary intake versus previous results in relation to nutrient levels may be that blood levels of many micronutrients, especially those readily sequestered during inflammation, do not very closely reflect current dietary intake [27].

The elderly in this study had relatively high adiposity. While obesity does not cause marked changes in immune function [28] it can accelerate thymic aging [29] and promote chronic inflammation [30]. In our study, a positive relationship was evident between body adiposity and the numbers of B cells but not T cells. Previous findings in institutionalised elderly women also found that the plasma B-cell count was related to adiposity (as assessed by bioelectrical impedance) [31]. It has been suggested that accumulating B cells may play a role in the development of obesity-related insulin resistance [32]. Additionally, murine studies suggest higher B cell numbers in the plasma of aged animals signal the development of autoimmunity that increases with aging [33].

The ratio of CD4^+^:CD8^+^ cells appeared unrelated to survival in our elderly population (Table 4). In previous studies the importance of the CD4^+^:CD8^+^ ratio as an indicator of survival also does not emerge clearly. Although some studies suggest that high ratios of CD4^+^:CD8^+^ cells are associated with better survival, particularly in those aged over 85 years [34]– [36] data also indicate that inverted CD4^+^:CD8^+^ ratios may reflect persistent cytomegalovirus (CMV) infection [37]. As this viral infection is not consistently present in aging, it is unsurprising that the relationship of inverted CD4^+^:CD8^+^ ratios with mortality has been absent in some cohorts [38], [39].

The strongest relationship to emerge in our study population between lymphocyte subsets and poor survival was a high ratio of NK to CD4^+^ T cells (Table 4). A high NK:CD4^+^ T cell ratio reflects both the decline of the adaptive immune system together with the relative expansion of the innate immune system, both known to occur during aging [40]. Thus overall numbers of NK cells often appear to increase with age [41] although their functionality may be reduced unless zinc status is optimal [17]. Concurrently, T cell levels may decline due to marked deterioration of the thymic environment and reduced output of naïve T cells, the poor ability of T cells in the periphery to proliferate and decreased levels of T cell survival [6], [7]. The NK:CD4^+^ T cell ratio is relatively easy to determine and if results are confirmed in further studies it may potentially have considerable clinical utility.

This study is limited by the small sample size, relatively short follow-up and the use of a convenience sample of volunteer participants. Moreover, these participants came from a developed country. Changes in lymphocyte populations with age may therefore differ from those found in developing countries with higher levels of malnutrition and infectious disease [42]. In addition, in our study immune status was judged by absolute and relative numbers of lymphocyte subsets only, measures of naïve or memory phenotypes were not assessed. Most importantly, measures of function were not determined. High cell numbers per se do not imply improved functionality. One study reported that low NK activity (as determined by a chromium release assay) but not NK cell numbers related to low survival times in Japanese elderly with high rates of infectious disease [43]. One strength of our study was that methods for data collection of weight, body composition and other measures of nutritional intake and status are all objectively based. Moreover, this is the first study to our knowledge to report on immunity and nutrition in Australian elderly residing in low-level care.

## Conclusions

This study in Australian institutionalised elderly has demonstrated a strong predictive relationship between poor survival and the ratio of NK-cells (markers of innate immunity) to CD4^+^ T cells (markers of adaptive immunity). Further study is needed to establish the utility of this ratio for predicting longevity in other elderly populations.

## Methods

Participants were elderly men and women from 14 low-level aged care facilities in metropolitan Melbourne, Australia. Low-level care provides accommodation, assistance with personal care and basic nursing care (e.g., medication and health monitoring) but does not provide 24 h nursing care.

The present study was nested within a larger cluster design, randomised control trial [14]. Participants were enrolled if they were ambulatory and able to self-feed. All were receiving assistance with activities such as personal care whether this was required or not. While 78 women and 25 men were recruited, only 64 women and 24 men for whom data on immune status were available are given here. Participants included did not differ in age, BMI, body composition, dietary intake, functional status or medical conditions and medications from those not included (data not shown). The study was approved by the Human Research Ethics Committee, Austin Health and by the Standing Committee on Ethics in Research involving Humans, Monash University.

Measurement of body height and weight, calculation of BMI, the determination of body composition by dual energy X-ray absorptiometry densitometry (DXA) and the calculation of %SMI were undertaken as described previously [44]. Sarcopenia was considered to be present if mean SMI% was >1SD below the mean SMI% of a young and apparently healthy reference group [44].

Trained dietitians collected 3-day records based on the weighed intake of all foods, beverages, and food supplements taken at main meals plus morning and afternoon tea with foods weighed to ±1 g on digital scales (Soehnle Venezia, Switzerland). A recall of foods and beverages consumed outside set meal times was also taken. Mean daily intake was calculated using SERVE Nutrition Management System version 5.0.012, 2004 (Serve Nutrition Systems, St Ives, NSW.) based on Australian food composition tables. Nutrient intakes were compared to Estimated Average Requirements (EAR) for Australia and New Zealand [45].

Non-fasting, peripheral venous blood was analysed for albumin at Network Pathology, Austin Health, Melbourne and high sensitivity CRP, IL-6, haemoglobin and ROS production within neutrophils were measured at Southern Cross Pathology, Monash Medical Centre, Melbourne, as previously described [14], [46].

Lymphocyte subsets were labeled and analysed by flow cytometry following methods described by King et al. [47]. The following subsets were identified: CD3^+^ (total T-cells), CD3^+^CD4^+^ (CD4^+^ T cells), CD3^+^CD8^+^ (CD8^+^ T cells), CD3^−^CD16^±^CD56^±^ (NK cells) and CD19^+^ (B-cells). Enumeration of these subsets, after addition of appropriate monoclonal antibodies and fluorescenated second antibody [47] was carried out using a Beckman-Coulter Q-prep machine flow cytometer (Hialeah, FL, USA) at the Monash Centre for Inflammatory Diseases, Monash University Department of Medicine, Monash Medical Centre, Melbourne. Total lymphocyte number, obtained from the full blood count, was used to compute the absolute numbers of lymphocytes for each subset.

Age (in years) was calculated as the difference between the date of first assessment and the reported date of birth. Comorbidity was defined as the number of current chronic conditions based on medical record report of cardiovascular disease, stroke, cancer, diabetes, Parkinson’s disease, kidney disease or lung disease. Long term medications were defined as current medications that were taken to treat a chronic condition. A registered nurse recorded disease conditions and medications and the occurrence of mortality from resident medical records maintained at each facility. These medical records were examined every 3 months throughout the 143 week follow-up period.

Data were analysed using SPSS for Windows (Version 19, SPSS, IBM). Descriptive data are given as the mean ± SD for continuous variables and where relevant, the prevalence of values below or above the reference range was determined. Differences between groups were tested using Student’s *t*-test for unpaired data while differences in proportion were compared with the chi-square test. Univariate associations with lymphocyte subsets were established using Pearson’s correlation. Associations of age, gender, medical conditions, body fat and serum albumin with lymphocyte subsets in peripheral blood were then examined using multivariate linear regression. Relationships between survival and lymphocyte subsets in blood were examined by Cox proportional regression and expressed as Hazard Ratios (HR) with 95% confidence intervals (CI). The assumption that hazards were proportional was assessed via log-minus-log survival plots. Significance was taken at *P* ≤ 0.05.

## Abbreviations

BMI: Body mass index; CD3+CD4+: CD4^+^ T cells; CD3+CD8+: CD8^+^ T cells; CI: Confidence interval; CRP: C-reactive protein; DXA: Dual energy X-ray absorptiometry; EAR: Estimated average requirements; HR: Hazard ratio; IL-6: Interleukin-6; NK: Natural killer; ROS: Reactive oxygen species; SD: Standard deviation; SMI%: Percent skeletal muscle.

## Competing interests

The authors declare that they have no competing interests.

## Authors’ contributions

JLW participated in study design, collected and analysed dietary data, organised lymphocyte subset analysis, performed all statistical analyses and drafted and finalised the manuscript. SI-B conceived the larger study design and made critical comments on the manuscript. KZW participated in the study design, data interpretation and contributed immunological expertise to the drafting and finalisation of the manuscript. All authors read and approved the final manuscript.

## Authors’ information

JLW (BSc, Grad Dip Nutrition and Dietetics, Grad Dip Health Promotion) is a dietitian and PhD candidate. S-IB (PhD, MNut) is Senior Research Officer. KZW (PhD, MND) is an Associate Professor and dietitian. Her PhD was in the field of immunology.
